# Bone marrow-derived extracellular vesicles carry the TGF-β signal transducer Smad2 to preserve hematopoietic stem cells in mice

**DOI:** 10.1038/s41420-023-01414-0

**Published:** 2023-04-05

**Authors:** Flavie Gautheron, Aleksandra Georgievski, Carmen Garrido, Ronan Quéré

**Affiliations:** 1 UMR1231, Inserm/Université Bourgogne, Dijon, France; 2 LipSTIC Labex, Dijon, France; 3 Centre Georges François Leclerc, Dijon, France

**Keywords:** Cell death, Haematopoietic stem cells

## Abstract

Extracellular vesicles (EVs) released by cells in the bone marrow (BM) are important for regulating proliferation, differentiation, and other processes in hematopoietic stem cells (HSC). TGF-β signaling is now well known to be involved in HSC’s quiescence and maintenance, but the TGF-β pathway related to EVs is still largely unknown in the hematopoietic system. We found that the EV inhibitor Calpeptin, when injected intravenously into mice, particularly affected the in vivo production of EVs carrying phosphorylated Smad2 (p-Smad2) in mouse BM. This was accompanied with an alteration in the quiescence and maintenance of murine HSC in vivo. EVs produced by murine mesenchymal stromal MS-5 cells also showed presence of p-Smad2 as a cargo. We treated MS-5 cells with the TGF-β inhibitor SB431542 in order to produce EVs lacking p-Smad2, and discovered that its presence was required for ex vivo maintenance of HSC. In conclusion, we revealed a new mechanism involving EVs produced in the mouse BM that transport bioactive phosphorylated Smad2 as a cargo to enhance the TGF-β signaling-mediated quiescence and maintenance of HSC.

## Introduction

Hematopoietic stem cells (HSC) reside at the top of the hematopoietic hierarchy and have capacities of self-renewal and differentiation, which are essential for the lifelong sustenance of the stem cell pool and the production of all types of blood cells. In the bone marrow (BM) microenvironment, HSC are supported by a large heterogeneous population of stromal cells in the perivascular niche, such as endothelial and mesenchymal cells, which generate signals regulating HSC self-renewal, quiescence, and differentiation [1, 2]. Cellular quiescence is a cell cycle arrest that is reversed in response to a combination of cell-intrinsic factors and environmental cues. In HSC, a coordinated balance between quiescence and differentiating proliferation ensures longevity [3]. Previous studies have shown that transforming growth factor beta (TGF-β) signaling is involved in HSC subtype modulation and quiescence, mainly by preventing HSC re-entry into the cell cycle [4–9]. The TGF-β ligands bind to TGF-β receptors (TGF-β-RI and TGF-β-RII) on the cell surface, causing their oligomerization and inducing activation of the protein kinase activity of the type I receptor. Once phosphorylated and activated, the substrate of the TGF-β-RI are Smad family member 2 and 3 (Smad2 and Smad3), which, upon phosphorylation, oligomerize with Smad4, accumulate in the nucleus and regulate gene transcription involved in HSC quiescence [4]. TGF-β is produced as a latent form by a variety of cells, and the nonmyelinating Schwann glial cells are components of the BM niche that maintain HSC hibernation by regulating activation of latent TGF-β [7].

Extracellular vesicles (EVs) implicated in further hematopoietic support have been shown to be mainly produced by the mesenchymal stromal cells (MSC) [10–20]. They are important for cell–cell communication, and the diverse mechanisms involve proteins or miRNAs that are carried by EVs with various roles in regulating proliferation, differentiation, or other properties of HSC homeostasis [10–20]. Studies have also shown that when regulatory molecules for TGF-β were detected in EVs, they induced downstream Smad2/3 signaling in targeted cells [21]. EVs derived from MSC isolated from human BM promoted the ex vivo expansion of HSC, and among cargoes overrepresented in EVs, the authors discovered several proteins of the TGF-β pathway, including Smad [12]. In this study, we discovered that EVs produced in the mouse BM environment contained phosphorylated Smad2, a bioactive component that transmits a TGF-β signal in cells [4]. We then addressed the hypothesis that EVs might carry this TGF-β signal transducer precisely for quiescence and maintenance of HSC.

## Results

### Treatment of mice with an EV inhibitor affects production of EVs carrying p-Smad2

**Fig. 1:**
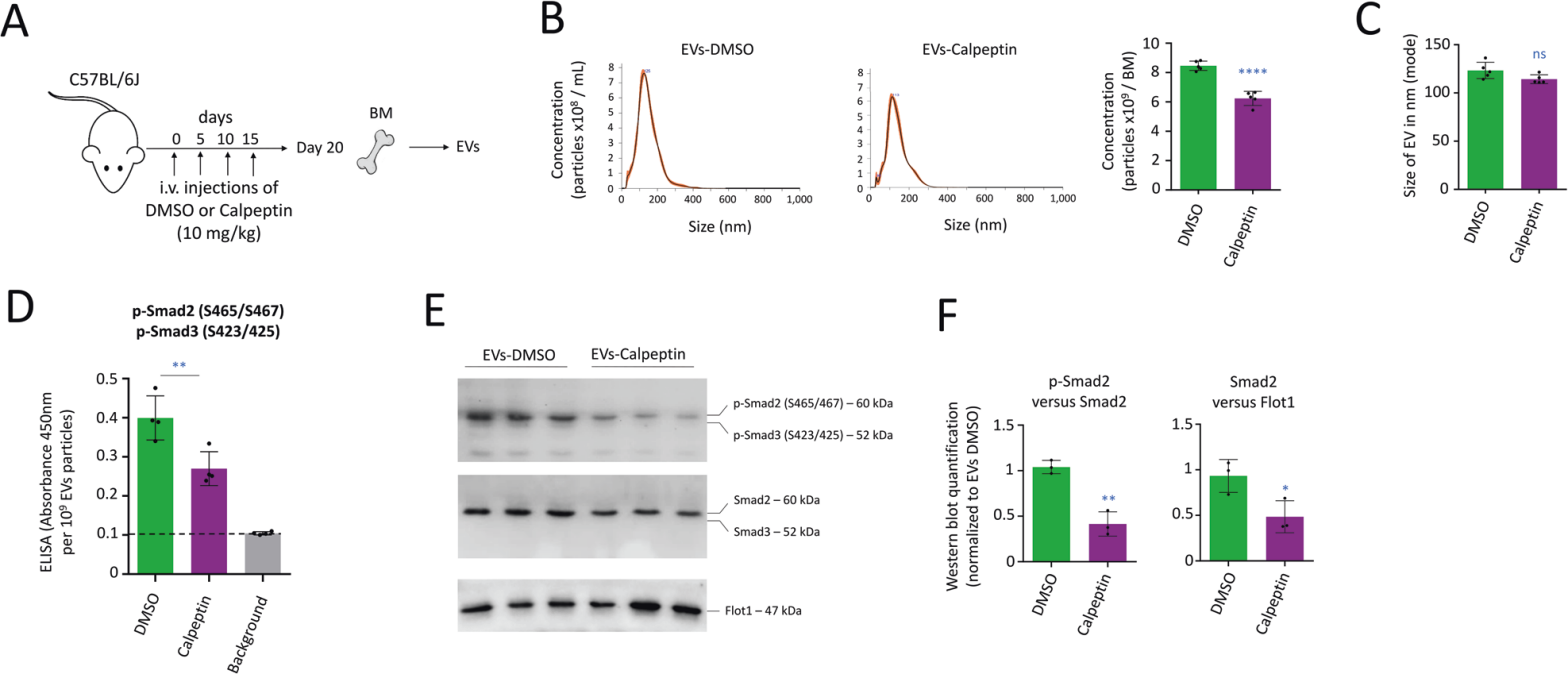
Treatment of mice with the EV inhibitor Calpeptin affects the in vivo production of EVs carrying p-Smad2. **A** Procedure followed to treat C57BL/6J mice with Calpeptin in order to analyze EVs in BM. **B** NTA quantification of EVs isolated from mice BM at day 20. Data showing that Calpeptin reduced the number of EVs in BM by ~26%, *n* = 5 mice per group. **C** Size of EVs measured by NTA. **D** ELISA quantification of EVs showing a reduction in the detection of p-Smad2/3. Quantification was normalized to 10^9^ particles, *n* = 4 mice per group. **E** Western blot with 50 µg of proteins extracted from EVs, *n* = 3 mice per group. Data showing the presence of p-Smad2 and Smad2, but not p-Smad3 and Smad3. Flotillin 1 (Flot1) generally detected on EVs was used as an endogenous control. **F** Quantification of the Western blots. On this figure, data are shown as means ± SD. *P* value measured by two-tailed unpaired Student’s *t* test; *P < 0.05; **P < 0.01; ****P < 0.0001; ns, non-significant.

In order to assess the effect of the inhibition of EV production on HSC in vivo, we intravenously (i.v.) injected C57BL/6J mice with Calpeptin, an inhibitor of calpains, injectable in vivo at 10 mg/kg [22]. Four administrations were performed every 5 days; 5 days after the last injection, tibias and femurs from the two bottom legs were crushed to isolate EVs from the BM (Fig. 1A). Nanoparticle tracking analysis (NTA) showed that the number of EVs produced in BM by mice treated with Calpeptin was reduced by ~26%, with 8.4 × 10^9^ and 6.2 × 10^9^ particles, respectively detected for control mice and Calpeptin-treated mice (*P* < 0.0001, Fig. 1B). NTA showed furthermore that treatment with Calpeptin did not affect the size of the EVs produced in BM (Fig. 1C).

Through enzyme-linked immunosorbent assay (ELISA), we detected that the EVs produced in mouse BM treated with Calpeptin showed a reduction in the level of p-Smad2/3 (*P* < 0.01, Fig. 1D). Since p-Smad2/3 are important downstream mediators involved in the TGF-β-dependent maintenance of HSC [4], we decided to study their presence with Western blot. We confirmed that EVs produced in the BM environment produced phosphorylated Smad2 (p-Smad2) on Serine’s S465/467 (Fig. 1E). We did not detect other proteins of the TGF-β pathway in EVs, such as Smad3 or phosphorylated Smad3 (on Serine’s S423/425), the TGF-β receptors RI and RII, as well as the co-Smad4 protein (Supplementary Fig. S1). Through quantification of the intensities of the proteins, we confirmed a reduction in p-Smad2 levels (*P* < 0.01), as well as the total quantity of Smad2 (*P* < 0.05) for Calpeptin-treated animals, compared with control mice (Fig. 1F).

In conclusion, treatments of mice with the EV inhibitor Calpeptin affected the quantity of EVs produced in the BM environment and also the presence of p-Smad2 in these EVs. This TGF-β signal transducer is a major component of the TGF-β pathway once phosphorylated, so we decided to analyze how primitive hematopoietic cells were altered in the BM environment following treatments with Calpeptin.

### Treatment of mice with Calpeptin affects HSC maintenance

**Fig. 2:**
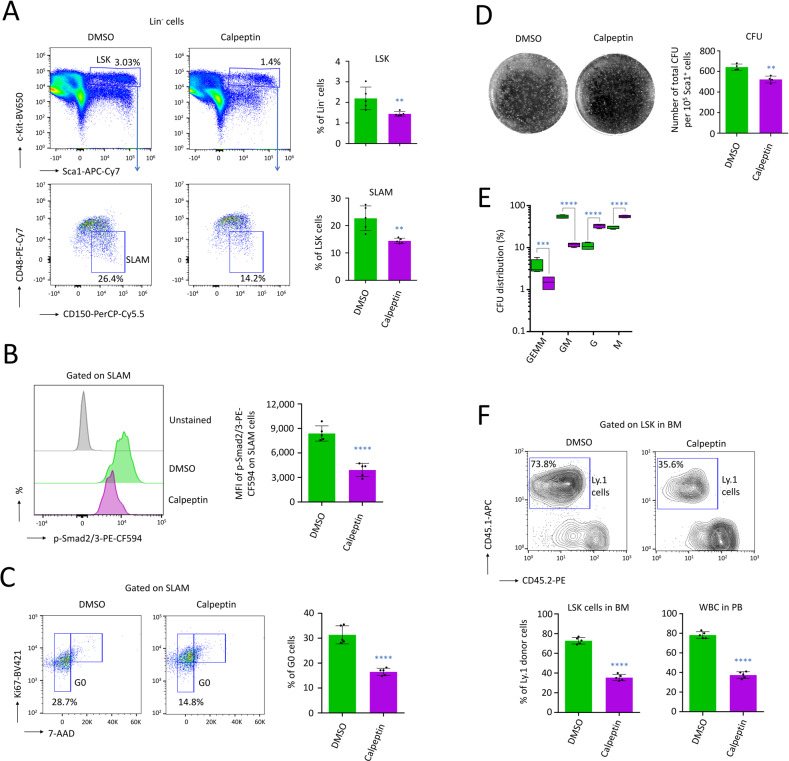
Treatment of mice with the EV inhibitor Calpeptin affects maintenance of HSC in vivo. **A** Flow cytometry on Lin^-^ cells showing that a treatment with Calpeptin decreased the percentage of hematopoietic progenitors (LSK cells) as well as HSC (SLAM cells), *n* = 5 mice per group. **B** Mean fluorescence intensity (MFI) measured by flow cytometry on HSC (SLAM cells), after cell permeabilization, showing a loss of p-Smad2/3 following treatment with Calpeptin, *n* = 5 mice per group. **C** Flow cytometry showing a reduction in quiescent HSC (in G0) following treatment with Calpeptin, *n* = 5 mice per group. **D** CFU assay on semi-solid media showing a reduction in the number of total CFU per 10^5^ Sca1^+^ cells, isolated from the BM of mice treated with Calpeptin. CFU were observed after 8 days of ex vivo culture, *n* = 4 mice per group. **E** Distribution among the CFU showing a reduction of the less differentiated progenitors (CFU-GEMM and CFU-GM), as well as an increase in mono-lineage progenitors (CFU-G and CFU-M) among Sca1^+^ cells isolated from the BM of mice treated with Calpeptin. **F** Analysis of the reconstitution capacity in vivo following the i.v. transplantation of 4 × 10^5^ Sca1^+^ cells freshly isolated from donor C57BL/6SJL (Ly.1) mice treated or not with Calpeptin, in C57BL/6J (Ly.2) recipient mice, *n* = 5 mice per group. Reconstitutions were assessed on WBC in PB, as well as on LSK cells after Lin^-^ depletion of BM cells, 16 weeks after transplantation. Examples of cytometry plots for LSK cells and statistics for LSK cells and WBC. On this figure, data are shown as means ± SD. *P* value measured by two-tailed unpaired Student’s *t* test; **P < 0.01; ***P < 0.001; ****P < 0.0001.

Following the same Calpeptin treatment described above, mouse tibias and femurs from the two bottom legs were crushed to isolate the primitive lineage negative (Lin^-^) BM cells. We detected a significant reduction among the hematopoietic stem and progenitor cells (HSPC, LSK; Lin^-^ Sca1^+^ c-Kit^+^ cells, *P* < 0.01), as well as HSC (SLAM; LSK CD48^-^ CD150^+^ cells, *P* < 0.01, Fig. 2A). Through flow cytometry, we also characterized a marked reduction of p-Smad2/3 (*P* < 0.0001) in HSC isolated from mice treated with Calpeptin (Fig. 2B). Importantly, Calpeptin did not modify p-Smad2/3 levels in HSC, and consequently it cannot be considered as an inhibitor of the TGF-β pathway (Supplementary Fig. S2). When we assessed the cell cycle activity, we observed that the HSC isolated from mice treated with Calpeptin were also showing lower percentage of cells in quiescence (*P* < 0.0001, Fig. 2C). When 10^5^ Sca1^+^ cells were cultured on semi-solid media, a lower number of the total hematopoietic colony-forming unit (CFU) was detected following eight days of culture (*P* < 0.01, Fig. 2D). When we assessed the CFU distribution, we observed a decrease in multipotent progenitors that give rise to erythroid, granulocyte-macrophage and megakaryocytic cells (CFU-GEMM, *P* < 0.001), or granulocyte-macrophage cells (CFU-GM, *P* < 0.0001). We also saw an increase in the mono-lineage progenitors that give rise to granulocytes (CFU-G, *P* < 0.0001) or macrophages (CFU-M, *P* < 0.0001, Fig. 2E). This experiment therefore suggests an exhaustion of the most primitive hematopoietic cells in vivo following treatment with the EV inhibitor.

Through the intravenous transplantation of Sca1^+^ cells isolated from donor C57BL/6SJL (Ly.1) mice into C57BL/6J (Ly.2) recipient mice, we detected a significant deficiency in hematological reconstitution when secondary mice were transplanted with Sca1^+^ cells treated with the Calpeptin inhibitor. Specifically, 16 weeks after the transplantation, we detected a significant reduction in the CD45.1 positive cells among LSK cells in BM (*P* < 0.0001), as well as among white blood cells (WBC) in peripheral blood (PB) (*P* < 0.0001, Fig. 2F).

In conclusion, when mice were treated with Calpeptin, we observed the expected reduction in the production of EVs in BM, and, unexpectedly, the levels of p-Smad2 were affected in these EVs. Treatment with Calpeptin was associated with a reduction in the p-Smad2/3 rates observed in HSC, as well as a loss of HSC quiescence and maintenance in vivo.

### Murine stromal MS-5 cells produce EVs carrying p-Smad2 in vitro

**Fig. 3:**
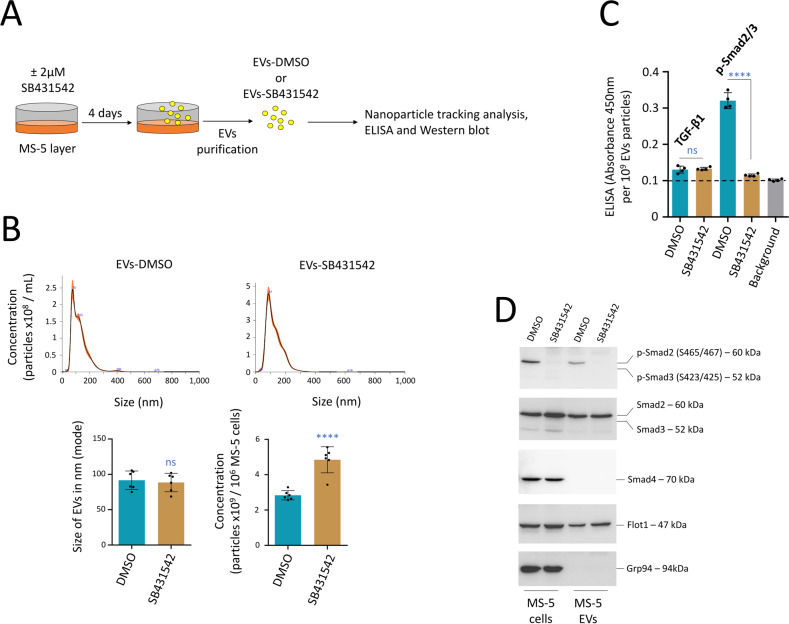
MS-5 cells produce EVs carrying p-Smad2 that are uptaken by HSC. **A** Procedure followed to produce EVs-DMSO or EVs-SB431542 with MS-5 cells. **B** NTA quantification and size of EVs isolated from the supernatant of MS-5 cells treated with the TGF-β type I receptor kinase (ALK5) inhibitor (EVs-SB431542) or not treated (EVs-DMSO). Data are shown as means ± SD, *n* = 6 biological samples. **C** ELISA showing the presence of p-Smad2/3 in MS-5 EVs. Treatment with SB431542 abrogated the production of p-Smad2/3 in EVs. Low production of the active TGF-β1 ligand in EVs was also detected by ELISA. Quantification was normalized to 10^9^ particles, *n* = 4 biological samples. **D** Western blot showing the production of p-Smad2 in MS-5 cells and EVs. The co-Smad4 protein was only detected in MS-5 cells, not in EVs. MS-5 cells showed an absence of p-Smad3, which was also not detected in EVs. Treatment with SB431542 abrogated the presence of p-Smad2, both in MS-5 cells and in EVs (EVs-SB431542). On this figure, data are shown as means ± SD. *P* value measured by two-tailed unpaired Student’s *t* test; ****P < 0.0001; ns, non-significant.

In the BM microenvironment, HSC are supported by MSC through direct cell-to-cell contact, cytokine secretion, and soluble growth factors [1, 2]. EVs implicated in further hematopoietic support are mainly produced by MSC [10–20]. The murine mesenchymal stromal MS-5 cells and their secreted molecules create a surrogate microenvironmental niche within which murine HSC and hematopoietic progenitors position themselves, allowing the regulation of differentiation or expansion in vitro [23, 24]. We developed a method to demonstrate that MS-5 cells produced EVs carrying p-Smad2 and to study the role mediated by the presence of p-Smad2 in EVs on HSC maintenance in vitro. We kept MS-5 cells in culture for four days in order to produce a sufficient quantity of EVs in the supernatant. To avoid contamination with unspecific EVs from serum, cells were maintained with a media depleted in EVs. Moreover, we treated MS-5 cells with the TGF-β type I receptor kinase (ALK5) inhibitor SB431542, used at 2 µM every day, during the four days of EV production (Fig. 3A). Media was recovered and pulled-down by successive centrifugations to remove residual cells and debris, and then EVs secreted in the MS-5 cell supernatant were recovered through ultra-centrifugation. NTA showed that while treatment with SB431542 did not affect the size of the EVs produced by MS-5 cells, the TGF-β inhibitor surprisingly increased the quantity of EVs produced in the media, with 2.8 × 10^9^ particles and 4.8 × 10^9^ particles per million of MS-5 cells, respectively, observed for DMSO control and SB431542 (Fig. 3B). Through ELISA, we established that SB431542 affected the presence of p-Smad2/3 in EVs, and the TGF-β1 ligand was weakly detected and unaffected by this treatment (Fig. 3C). Through Western blot, we confirmed that EVs produced by MS-5 cells showed presence of phosphorylated Smad2 (p-Smad2, on Serine’s S465/467) as well as the total Smad2 protein (EVs-DMSO), whereas we did not detect Smad3 or Smad4 in these EVs. Importantly, treatment with SB431542 abrogated the presence of p-Smad2, both in MS-5 cells and in EVs (EVs-SB431542) (Fig. 3D and Supplementary Fig. S3).

In conclusion, MS-5 cells produced EVs showing presence of the p-Smad2 effector. Following treatment of MS-5 cells with an inhibitor of the TGF-β type I receptor kinase (ALK5) which control the phosphorylation of Smad2, we generated EVs that did not produce p-Smad2.

### Murine stromal MS-5 cells produce EVs that can be uptaken by HSC ex vivo

**Fig. 4:**
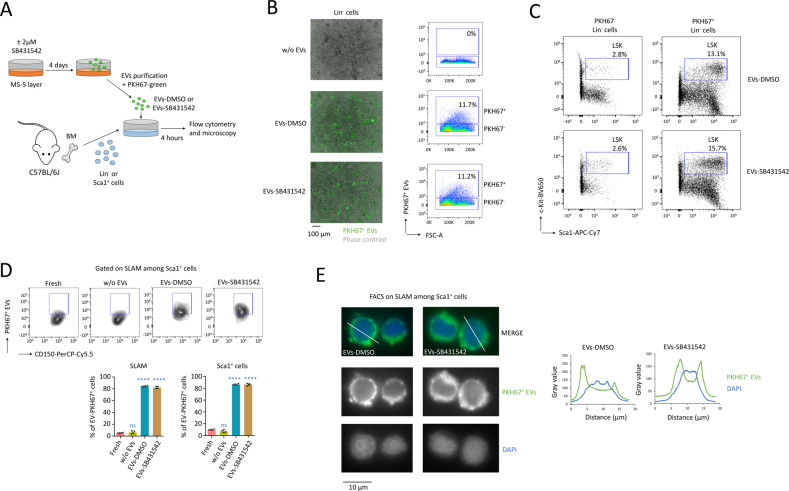
MS-5 cells produce EVs carrying p-Smad2 that can be uptaken by HSC. **A** Procedure followed to treat Lin^-^ and Sca1^+^ cells with PKH67^+^ EVs-DMSO or EVs-SB431542. We administered 10^9^ particles per 10^6^ Lin^-^ cells, or 10^9^ particles per 4 × 10^5^ Sca1^+^ cells. **B** Microscopy on a 96-well round bottom plate and flow cytometry, after the exposure of Lin^-^ cells during four hours with PKH67^+^ EVs. Data showing that ~11% of the Lin^-^ cells bound PKH67^+^ EVs. Magnification ×4, black scale bar corresponds to 100 µm. **C** Flow cytometry on PKH67^-^ and PKH67^+^ gated Lin^-^ cells showing that PKH67^+^ EVs bound more to HSPC (LSK; Lin^-^ Sca1^+^ c-Kit^+^ cells). **D** Sca1^+^ cells were freshly isolated from the BM of mice (fresh) and treated with EVs-DMSO, EVs-SB431542 or without EVs (w/o EVs) for four hours. Flow cytometry showing the uptake of PKH67^+^ EVs by >80% of the Sca1^+^ c-Kit^+^ cells, as well as >80% of HSC (SLAM cells). Data are shown as means ± SD, *n* = 4 biological samples. *P* value calculated against the fresh conditions and measured by one-way ANOVA with Tukey’s multiple comparison test; ****P < 0.0001; ns, non-significant. **E** Fluorescent optical sections, showing the uptake of PKH67^+^ EVs in the cytoplasm and nucleus of HSC (SLAM cells), purified by FACS, four hours after incubation with EVs-DMSO or EVs-SB431542. Magnification ×63, black scale bar corresponds to 10 µm, white bars correspond to the sections for measurement of the PKH67 green fluorescence and DAPI.

In order to make trackable EVs, we incubated the particles produced by MS-5 cells with the green fluorescent membrane labeling PKH67 dye just before their isolation by ultra-centrifugation. Using magnetic beads, we isolated the primitive Lin^-^ or Sca1^+^ cells from the BM of C57BL/6J mice, which were then incubated with PKH67^+^ EVs produced by MS-5 cells (Fig. 4A). Following 4 h of exposure ex vivo, we observed, by microscopy and flow cytometry, uptake of PKH67^+^ EVs-DMSO or EVs-SB431542 by ~11% of the Lin^-^ cells (Fig. 4B). Furthermore, the primitive HSPC (LSK; Lin^-^ Sca1^+^ c-Kit^+^ cells) were binding PKH67^+^ EVs (Fig. 4C). Through flow cytometry, we detected that >80% of the Sca1^+^ c-Kit^+^ cells, as well as HSC (SLAM; Sca1^+^ c-Kit^+^ CD48^-^ CD150^+^ cells) had uptaken PKH67^+^ EVs-DMSO or EVs-SB431542 following 4 h of exposure ex vivo (*P* < 0.0001, Fig. 4D). This uptake was also observed by fluorescence microscopy in the cytoplasm and nucleus of HSC (Fig. 4E).

In conclusion, when BM cells were treated with these EVs for 4 h in vitro, primitive hematopoietic cells, such as Sca1^+^ progenitors, as well as HSC (SLAM) had uptaken MS-5 EVs. We therefore went on to study the impact of murine MS-5 stromal EVs deprived of p-Smad2 on the maintenance of HSC ex vivo.

### MS-5-EVs carrying p-Smad2 maintains quiescence of HSC ex vivo

**Fig. 5:**
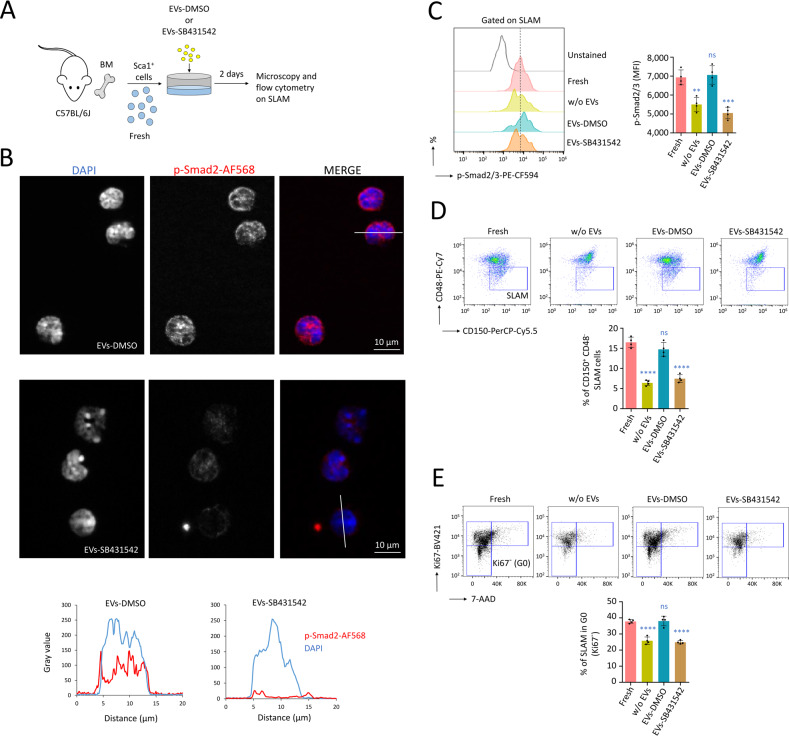
MS-5-EVs carrying p-Smad2 allow maintenance of HSC ex vivo. **A** Procedure followed to maintain Sca1^+^ HSC freshly (fresh) isolated ex vivo, with MS-5-EVs carrying p-Smad2 or not due to a treatment of MS-5 cells with SB431542. We administered 10^9^ particles per 4 × 10^5^ Sca1^+^ cells for 48 h. **B** Fluorescent optical sections, showing high level of p-Smad2 in the nucleus of HSC after incubation with EVs-DMSO, while HSC treated with EVs-SB431542 showed a low intracellular level of p-Smad2. Magnification ×63, white scale bar corresponds to 10 µm, white bars correspond to the sections for measurement of p-Smad2 and DAPI fluorescence. PKH67^+^ SLAM cells were purified by FACS. **C** Flow cytometry on HSC (SLAM cells) after permeabilization showing that treatment with EVs-DMSO maintained p-Smad2/3 levels, while a decrease in active TGF-β signaling was observed with EVs-SB431542, as well as without EVs (w/o EVs). Data normalized to the same number of HSC. **D** Treatment with EVs-DMSO maintained the number of HSC (SLAM cells) after 48 h, while EVs-SB431542 induced their exhaustion. **E** Cell cycling activity of HSC, measured by flow cytometry after permeabilization, showing maintenance of quiescent HSC (Ki67^-^ SLAM cells in G0) following treatment with EVs-DMSO, but not with EVs-SB431542. In this figure, data are shown as means ± SD, *n* = 4 mice. *P* value calculated against the fresh condition and measured by one-way ANOVA with Tukey’s multiple comparison test; **P < 0.01; ***P < 0.001; ****P < 0.0001; ns, non-significant.

Sca1^+^ cells, freshly (fresh) isolated ex vivo from the BM of mice were maintained during 48 h ex vivo without EVs (w/o EVs), or under incubation with EVs-DMSO or EVs-SB431542 (Fig. 5A). Following intracellular staining with a p-Smad2 antibody and fluorescent microscopy, we observed that HSC (SLAM cells) incubated with EVs-DMSO showed high levels of p-Smad2 in the nucleus compared with HSC incubated with EVs-SB431542 (Fig. 5B). Following intracellular staining and flow cytometry, we observed that a treatment with EVs-DMSO preserved the p-Smad2/3 level (*P* > 0.5, compared with fresh condition), whereas the level of p-Smad2/3 decreased significantly following treatment with EVs-SB431542 (*P* < 0.001, compared with fresh conditions, Fig. 5C). We then observed that a treatment with EVs-DMSO for 48 h ex vivo preserved HSC numbers (SLAM cells) at a level which was similar to HSC freshly isolated from mice (*P* > 0.05 between both conditions). However, this maintenance was not conserved with EVs-SB431542 (*P* < 0.0001, compared with fresh condition, Fig. 5D). When we assessed cell cycle activity, we observed that HSC treated with EVs-DMSO showed a higher percentage of cells in quiescence (Ki67^-^ SLAM cells in G0). On the contrary, the percentage of Ki67^+^ cells was increased in cells treated with EVs-SB431542 (Fig. 5E). Indeed, when Sca1^+^ cells were treated with EVs that did not contain p-Smad2, a loss in HSC maintenance was observed ex vivo.

**Fig. 6:**
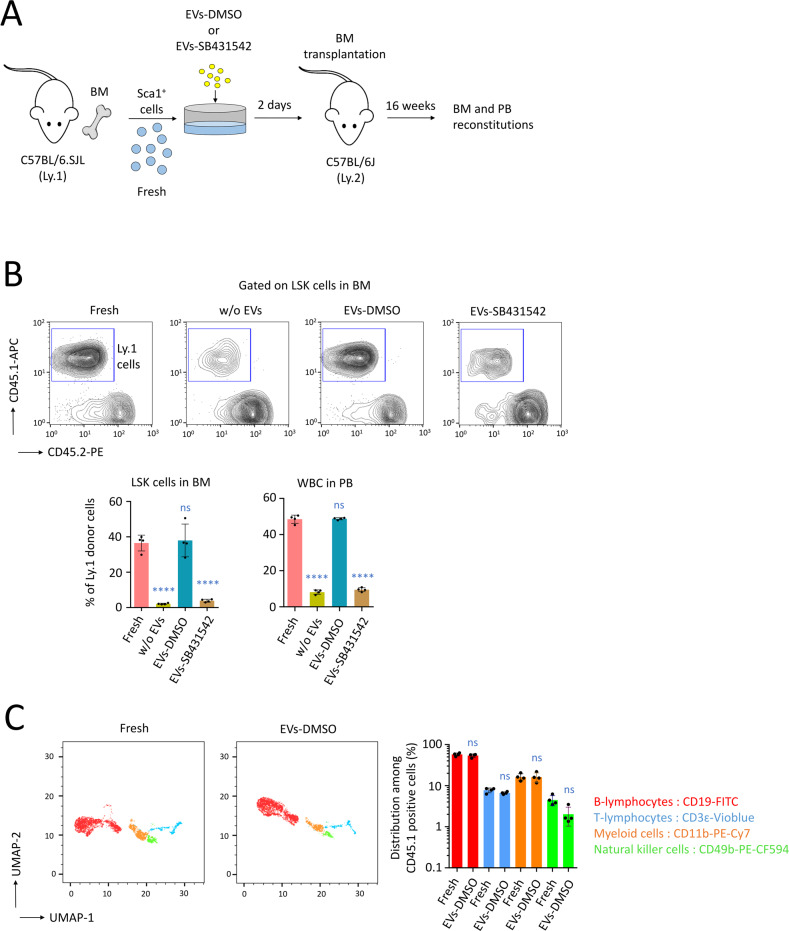
Transplantation of HSC, maintained ex vivo with MS-5-EVs carrying p-Smad2. **A** Procedure followed to maintain Sca1^+^ HSC ex vivo for 48 h with EVs-DMSO or EVs-SB431542. **B** Analysis of the reconstitution capacity in vivo following the i.v. transplantation of 4 × 10^5^ Sca1^+^ cells in C57BL/6J (Ly.2) recipients, *n* = 4 mice per group. Sca1^+^ cells isolated from donor C57BL/6SJL (Ly.1) mice were freshly injected (fresh) or exposed to EVs ex vivo for 48 h before the transplantation. We assessed the reconstitution 16 weeks after the transplantation on LSK cells in BM, as well as on WBC in PB. Examples of cytometry plots for LSK cells, statistics on LSK cells, and WBC. Data are shown as means ± SD, *n* = 4 mice. *P* value calculated against the fresh condition and measured by one-way ANOVA with Tukey’s multiple comparison test; ****P < 0.0001; ns, non-significant. **C** UMAP data frame showing distribution among CD45.1 positive WBC in different hematological lineages (B-lymphocytes, T-lymphocytes, myeloid cells, and natural killer cells). Data showing that Sca1^+^ cells treated ex vivo with EVs-DMSO for 48 h were able to reconstitute hematopoiesis in all lineages in secondary recipient mice, and to a level that was similar to fresh Sca1^+^ cells. Data are shown as means ± SD, *n* = 4 mice. *P* value measured by two-tailed unpaired Student’s *t* test; ns, non-significant.

The subsequent analysis of the reconstitution capacity in vivo was performed in C57BL/6J (Ly.2) recipients following the intravenous transplantation of Sca1^+^ cells freshly isolated from donor C57BL/6SJL (Ly.1) mice or exposed to EVs in vitro for 48 h (Fig. 6A). Sixteen weeks after the transplantation, mice transplanted with 4 × 10^5^ Sca1^+^ cells exposed to EVs-DMSO showed hematopoietic reconstitution similar to those who received fresh Sca1^+^ cells as assessed by comparable CD45.1 staining of LSK cells (*P* > 0.5) measured by flow cytometry in BM, and WBC analysis in PB (Fig. 6B). On the contrary, a low reconstitution capacity was detected in BM and PB when Sca1^+^ cells were not treated with EVs or exposed to EVs-SB431542 (*P* < 0.0001, compared with the fresh condition). We also confirmed that Sca1^+^ cells exposed to EVs-DMSO for 48 h ex vivo have the same capacity as Sca1^+^ cells freshly isolated from Ly.1 mice to reconstitute in all lineages of hematopoiesis (B- and T-lymphocytes, myeloid cells as well as natural killer cells, Fig. 6C and Supplementary Fig. S4).

In conclusion, following the treatment of MS-5 cells with TGF-β type I receptor kinase (ALK5) to prepare EVs deprived of p-Smad2, we demonstrated that this TGF-β effector was required in EVs to preserve quiescence and maintenance of HSC ex vivo.

## Discussion

EVs have a critical role in cancer development through interactions with the tumor microenvironment [21, 25–27]. Several molecules, including mRNAs, non-coding RNAs, and proteins known to be associated with the TGF-β pathway, have been reported as constituents among cargoes in solid cancer-derived EVs [21]. The presence of TGF-β-related proteins as EVs cargoes has been established in cancer-associated fibroblast, epithelial to mesenchymal transition, as well as cancer metastasis [21]. Furthermore, EVs released from leukemia cells are responsible for the suppression of HSC functions through stromal reprogramming of niche-retention factors or as a consequence of leukemia EV-directed carrier delivery to HSC [28–34]. However, the presence of TGF-β-related proteins among leukemia-derived EVs cargoes has not yet been reported.

In the normal BM niche, HSC reside at the top of the hematopoietic hierarchy and can self-renew and differentiate, which are essential processes for the lifelong sustenance of the stem cell pool and the production of all types of blood cells. Several studies have shown that TGF-β signaling is involved in HSC quiescence by preventing the re-entry of HSC into the cell cycle [4–9]. TGF-β is produced as a latent form by supported cells in the mouse BM microenvironment, and the nonmyelinating Schwann glial cells are a component of the BM niche that was found to be responsible for the maintenance of HSC hibernation by regulating latent TGF-β activation [7]. In the BM microenvironment, HSC were supported by MSC that generate signals regulating HSC self-renewal and the coordinated balance between quiescence and differentiation [1–3]. Interestingly, a study described how EVs produced by MS-5 cells can protect Sca1^+^ cells from lethal irradiation, although further investigations are needed to fully explore the role of EV cargo in mediating this protective effect [35]. Recent studies have demonstrated that MSC-EVs play an influential role in BM niches because of their unprecedented capacity to regulate the fate of HSC [20]. While many studies have demonstrated that some EVs in the normal BM niche communicate between MSC and HSC [12–20], only one study has described the presence of TGF-β-related proteins among cargoes in EVs produced by adult MSC [12]. Studies have also shown that when regulatory molecules for TGF-β are detected in EVs, they often induce downstream Smad2/3 signaling in targeted cells [21]. EVs derived from human MSC have been shown to promote the ex vivo expansion of HSC, and, interestingly, proteins involved in the TGF-β pathway have also been identified among cargoes in these EVs [12]. In our study, we revealed the existence of phosphorylated Smad2 in EVs isolated ex vivo from the mouse BM microenvironment. We also found that EVs produced in vitro by murine mesenchymal stromal MS-5 cells contained phosphorylated Smad2. Nevertheless, for an optimal ex vivo expansion of HSC, administration of recombinant TGF-β1 ligand was more efficient than the use of EVs (Supplementary Fig. S5). Consequently, treatment with preconditioned MSC-EVs should be improved in view of a future novel approach to ex vivo HSC maintenance for transplantation.

Here, we discovered that EVs released by murine MSC in vitro, as well as in the in vivo BM microenvironment, contained the phosphorylated Smad2 signal transducer, an important effector of TGF-β signaling involved in the homeostasis of HSC [4–9]. When mice were treated with the EV inhibitor Calpeptin, the quantity of EVs produced in the BM environment was altered, as was the presence of p-Smad2 in EVs. These observations were accompanied by a reduction in the levels of p-Smad2/3 in HSC, as well as a loss of HSC quiescence and maintenance in vivo. Following the inhibition of TGF-β type I receptor kinase (ALK5) to prepare EVs deprived of p-Smad2, we demonstrated that this TGF-β effector in EVs was required to preserve the p-Smad2/3 rates observed in HSC, and their quiescence and maintenance ex vivo. We also observed that treatment with this inhibitor increased the number of EVs produced by MS-5 cells. Therefore, it might be interesting to study the role mediated by the TGF-β pathway in the regulation of EVs produced by MSC.

**Fig. 7:**
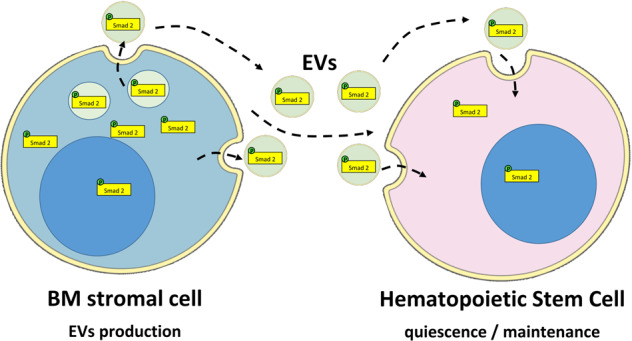
BM-derived EVs carry the TGF-β signal transducer Smad2 for the homeostasis of HSC. Schematic illustration of the proposed mechanism involving EVs produced by MSC that carry the cargo p-Smad2 involved in the quiescence and maintenance of HSC.

In conclusion, this study reveals a new mechanism involving EVs produced by supportive cells in the mouse BM microenvironment. These EVs transport bioactive phosphorylated Smad2 as a cargo, enhancing TGF-β signaling-mediated quiescence and maintenance of HSC (Fig. 7). The role of EVs in delivering bioactive TGF-β signaling to neighboring cells is an unexplored area, and our data highlights a new mode of regulation of stem cell behavior that could potentially be modulated for ex vivo expansion of these important cells.

## Materials and methods

### Treatment of mice with Calpeptin

The ethics committee for animal welfare of the University of Burgundy and the French ministry of higher education and research approved all animal experiments (under reference APAFIS #36855-2022041914584260). Calpeptin (C8999, Merck) was reconstituted in DMSO at 10 mg/mL. C57BL/6J (Ly.2) (Envigo) or C57BL/6SJL (Ly.1) mice (Charles River Laboratories) were i.v. injected with Calpeptin at 10 mg/kg, receiving four administrations every 5 days. Male and female mice, 7–16-week-old, were randomly allocated to experimental groups and no blinding method was used for experiments. There were no animal exclusion criteria. Five days after the last injection, bones (tibias and femurs) from the two bottom legs were crushed in a mortar in 2 mL of PBS1×. After centrifugation at 500 × *g* for 5 min, the supernatant was recovered to purify EVs following the procedure described below. Cells were washed in 10 mL PBS1× and filtrated with a sterile cell strainer at 70 µm, after centrifugation, Lin^-^ cells (130-110-470, Miltenyi Biotec) or Sca1^+^ cells (130-123-124, Miltenyi Biotec) were magnetically purified using LS columns (Miltenyi Biotec), counted with Trypan blue (Thermo Fisher Scientific) and processed with flow cytometry procedure, transplantation or CFU assay.

### Purification of EVs from mice

The supernatant was recovered for another centrifugation (10,000 × *g*, 5 min) to remove cell debris. The supernatant was then recuperated, and we pulled-down EVs by using the total EV isolation kit (15254394, Invitrogen) used at 10% (v/v), mixed by inverting the tubes several times and incubated for 15 min at 4 °C followed by centrifugation (10,000 × *g*, 10 min). The pellet of EVs was reconstituted in 100 µl of 0.1 µm-filtrated PBS1×. EVs were evaluated for their size and concentration by nanoparticle tracking analysis (NTA) using a NanoSight NS300 Instrument (Malvern Instruments) and conserved at −80 °C. EVs were suspended in lysis buffer (Cell Signaling Technology) and characterized by enzyme-linked immunosorbent assay (ELISA) for p-Smad2 (S465/S467)/p-Smad3 (S423/S425) (12001C, Cell Signaling Technology) or active TGF-β1 (437707, Biolegend).

### Purification of EVs from murine MS-5 cells

Murine MS-5 mesenchymal stromal cells (ACC-441, DSMZ) were cultured in Iscove’s Modified Dulbecco’s Media (IMDM) (Thermo Fisher Scientific), supplemented with 10% fetal bovine serum (FBS) and 1% Penicillin-Streptomycin-Amphotericin (PSA) (Pan Biotech, Aidenbach, Germany). When cell confluence in a T75 flask reached 80%, we administered IMDM supplemented with 20% of EV-depleted FBS and 1% PSA. To deplete EVs from FBS, we ultra-centrifuged at 120,000 × *g* (Optima XE-90 ultracentrifuge, Beckman Coulter) overnight in Ultra-Clear centrifuge tubes (Beckman Coulter). MS-5 cells were treated with the TGF-β-RI inhibitor SB431542 (S4317, Merck), used at 2 µM each day, for four days. Then, we recovered the supernatant (30 mL), cells were removed after centrifugation (500 *×* *g*, 5 min) and the supernatant was recovered for another centrifugation (10,000 × *g*, 10 min) to remove cell debris. The supernatant was recovered and we pulled-down EVs by ultra-centrifugation at 120,000 × *g* during 90 min. For microscopy, 20 µl of PKH67 green fluorescent cell linker kit for general cell membrane labeling (Merck) was administered for 30 min in the supernatant and we pulled-down EVs by ultra-centrifugation at 120,000 × *g* during 90 min. EVs in the pellet were reconstituted in 0.1 µm-filtrated PBS1× and evaluated for their size and concentration by NTA using a NanoSight NS300 Instrument (Malvern Instruments, Malvern, England) and conserved at −80 °C. For Western blot and ELISA, EVs were directly reconstituted in lysis buffer (Cell Signaling Technology).

### MS-5 EVs tested on primitive hematopoietic cells ex vivo

Tibias and femurs from the two bottom legs of C57BL/6J mice (Envigo) were crushed in a mortar in 10 mL of PBS1×. Total BM cells were then filtered with a sterile cell strainer at 70 µm, and Lin^-^ cells (130-110-470, Miltenyi Biotec) or Sca1^+^ cells (130-123-124, Miltenyi Biotec) were magnetically purified using LS columns (Miltenyi Biotec). Sca1^+^ cells were divided into four wells (fresh, w/o EVs, EVs-DMSO and EVs-SB431542) and treated with 10^9^ particles per 4 × 10^5^ cells. Lin^-^ cells were divided in three conditions (w/o EVs, EVs-DMSO and EVs-SB431542) and treated with 10^9^ particles per 10^6^ cells. To determine cell uptake of EVs, PKH67^+^ EVs were applied to cells for 4 h. Using flow cytometry or microscopy, we determined the percentage of Lin^-^, Sca1^+^ or SLAM cells that had uptaken fluorescent PKH67^+^ EVs. EVs and cells were co-cultured for 48 h in 96-well round bottom plates with 200 µL of the StemMACS media (Miltenyi Biotec), supplemented with PSA, murine Stem cell factor (SCF, 25 ng/mL, 130-101-741, Miltenyi Biotec), murine Interleukin 3 (IL3, 10 ng/mL, 130-096-687, Miltenyi Biotec) and murine Interleukin 6 (IL6, 10 ng/mL, 130-096-682, Miltenyi Biotec). Recombinant TGF-β1 ligand (Miltenyi Biotec) was used at 10 ng/mL for 48 h using the same culture conditions. By flow cytometry, we tested the effect of 48 h of exposure of Sca1^+^ cells to EVs, on the cell cycle activity of SLAM cells, following Ki67 staining. We also tested activation of the TGF-β pathway, following p-Smad2/3 staining. To assure that Calpeptin was not an inhibitor of the TGF-β pathway, we treated 2.5 × 10^5^ Lin^-^ cells ex vivo with 0.5 µM of SB431542 or 0.5 µM of Calpeptin for 18 h, then we assessed p-Smad2/3 by flow cytometry on SLAM cells.

### Transplantation of primitive hematopoietic cells

Sca1^+^ cells, either freshly isolated from the BM of C57BL/6.SJL (Ly.1) mice (Charles River Laboratories), or after their exposure to EVs for 48 h, were quantified by blue Trypan. Then, 4 × 10^5^ cells were i.v. transplanted in the tail vein of C57BL/6J (Ly.2) recipient mice (Envigo), pretreated, one and two days before the transplantation, with intraperitoneal injections of 20 mg/kg of Busulfan (Merck). Sixteen weeks after the transplantation, the percentages of donor cells were quantified in PB after hemolysis, as well as in BM, using flow cytometry. Donor Sca1^+^ cells (4 × 10^5^ cells) isolated from Ly.1 mice treated with Calpeptin were also transplanted in Ly.2 recipient mice, and hematopoiesis reconstitution was monitored by flow cytometry, 16 weeks after the transplantation, in the BM and PB of recipient mice.

### Hematopoietic colony-forming unit (CFU) assay

Sca1^+^ cells (130-110-470, Miltenyi Biotec) were isolated from mice treated with Calpeptin and counted, and 10^5^ cells were added to semi-solid methylcellulose media supplemented with growth factors (Methocult M3434; Stem Cell Technologies) at 37 °C in 98% humidity and 5% CO_2_. Hematopoietic CFUs were counted after eight days. Total CFU and distribution among CFU-GEMM, CFU-GM, CFU-G, and CFU-M were measured using a microscope.

### Fluorescent microscopy

Four hours following the incubation of Sca1^+^ cells with PKH67^+^ EVs (10^9^ particles per 4 × 10^5^ cells) in the StemMACS media (Miltenyi Biotec), cells were stained with cell surface markers (SLAM). Cells were fixed and permeabilized with BD Cytofix/Cytoperm Plus Fixation/Permeabilization Kit and Permeabilization Buffer Plus (BD Biosciences). We used the p-Smad2 (S465/467) antibody (#18338, Cell Signaling Technology) and the secondary anti-Rabbit-AF568 antibody (Thermo Fisher Scientific). Then, SLAM cells were purified by FACS and applied on a glass slide (Superfrost plus, Thermo Fisher Scientific) for up to 10 min and fixed with ProLong Gold Antifade reagent containing DAPI (P36931, Thermo Fisher Scientific). Fluorescent images were acquired with an Axio Imager M2 (Zeiss). Fluorescent optical sections of cells were obtained under magnification ×63 using an Axio Imager M2 (Zeiss) coupled with an Apotome.2 (Zeiss). Fluorescent images were processed for study (Fiji, NIH software).

### Western blot

Cells and EVs were suspended in Western blot lysis buffer (Cell Signaling Technology). On lysates, OD 620 nm was measured to normalize the amount of the loaded sample. 50 µg of proteins were supplemented with the Laemmli buffer (Cell Signaling Technology). Targeted proteins were separated on 10% SDS-PAGE gels and transferred to PVDF membranes. We used the anti-phospho-Smad2 (S465/467)/Smad3 (S423/425) antibody (1:1000, #8828, Cell Signaling Technology), a mix of the anti-Smad2 and anti-Smad3 antibodies (1:1000 each, #5339, #9523, Cell Signaling Technology), the anti-Smad4 antibody (1: 1000, #46535, Cell Signaling Technology), anti-Grp94 antibody (1:1000, ADISPA-850, Enzo) and the anti-Flot1 antibody (1:1000, 610821, BD Biosciences) used as a loading control. We also used anti-TGF-β-RI (ABF17-I, Merck) and anti-TGF-β-RII (MAB532, R&D Systems) antibodies. Appropriate secondary anti-rat, anti-mouse or anti-rabbit antibodies conjugated with Horseradish Peroxidase were used (1:5000, Cell Signaling Technology). Chemiluminescence was performed (Chemidoc, Bio-Rad) after applying ultra-sensitive enhanced chemiluminescent (ECL) substrate (SuperSignal West Femto Maximum Sensitivity, Thermo Fisher Scientific). Protein sizes were controlled with a protein ladder (Page Ruler Plus Prestained Protein Ladder, Thermo Fisher Scientific), and protein levels were assessed using ImageJ (NIH Software).

### Flow cytometry and fluorescent-activated cell sorting (FACS)

To stain hematopoietic populations on BM cells, we used the following antibodies on Lin^-^ purified cells (130-110-470, Miltenyi Biotec): Sca1-APC-Cy7 (1:100, 108126, Biolegend), c-Kit-BV650 (1:100, 135125, Biolegend), CD48-PE-Cy7 (1:200, 560731, BD Biosciences), CD150-PerCP-Cy5.5 (1:100, 115921, Biolegend). For PB reconstitution in vivo, we used the following antibodies: anti-CD45.1-APC antibody (1:100, 130-121-215, Miltenyi Biotec), anti-CD45.2-PE antibody (1:100, 130-124-080, Miltenyi Biotec), anti-CD49b-PE-CF594 (1:100, 562453, BD Biosciences), anti-CD11b-PE-Cy7 (1:100, 552850, BD Biosciences), anti-CD19-FITC (130-102-494, Miltenyi Biotec) and anti-CD3ɛ-Vioblue (1:100, 130-102-441, Miltenyi Biotec). Viability was assessed with Fixable Viability Stain FVS440UV, FVS450 or FVS780 (1:1000, BD Biosciences). After staining with cell surface markers and FVS for viability, cells were fixed and permeabilized using BD Cytofix/Cytoperm Plus Fixation/ Permeabilization Kit and Permeabilization Buffer Plus (BD Biosciences). For cell cycle studies, we used anti-Ki67-BV421 antibody (1:50, 562899, BD Biosciences) and 7-AAD (1:20, 559925, BD Biosciences). Anti-p-Smad2 (S465/S467)/p-Smad3 (S423/S425)-PE-CF594 antibody (1:100, 562697, BD Biosciences) was used after cell surface staining and fixation/permeabilization. Cell subsets were analyzed using Aurora (Cytek) or LSR-Fortessa (BD Biosciences). For fluorescent microscopy, SLAM cells were sorted on a FACS Aria cell sorter (BD Biosciences). Data were analyzed using FlowJo software (v10, TreeStar Inc, Ashland, USA). We used the Uniform Manifold Approximation and Projection (UMAP) FlowJo plugin for dimensionality reduction to visualize high parameter datasets in a two-dimensional space.

### Statistics

All data are expressed as means ± standard deviation (SD). Differences between two groups were assessed with the two-tailed Student’s unpaired *t* test. Differences between more than two groups were assessed with the one-way ANOVA with Tukey’s multiple comparison test. No statistical methods were used to predetermine the sample size. Mice were randomly allocated to experimental groups. No blinding method was used for injection. There was no animal exclusion criteria. The variance was similar between the groups that were being statistically compared. Statistics were performed using Prism 6 (GraphPad, San Diego, USA).

## Supplementary information

Supplementary Figures S1 to S5

## Data Availability

Data that support the findings of this study are available from the corresponding author on reasonable request.
