# Supplementary material for: Bone marrow-derived extracellular vesicles carry the TGF-β signal transducer Smad2 to preserve hematopoietic stem cells in mice
Source: Cell Death Discov. 2023 Apr 5;9:117. doi: 10.1038/s41420-023-01414-0 (PMC10076352; doi:10.1038/s41420-023-01414-0)
Supplement: Supplementary file 1 — Supplementary Figures S1 to S5 [file 41420_2023_1414_MOESM1_ESM.docx]

**Bone marrow-derived extracellular vesicles carry the TGF-β signal transducer Smad2** **to preserve hematopoietic stem cells in mice**

Flavie Gautheron,^1^ Aleksandra Georgievski,^1,2^ Carmen Garrido,^1,2,3^ and Ronan Quéré^1,2,*^

^1^UMR1231, Inserm/Université Bourgogne, Dijon, France

^2^LipSTIC Labex, Dijon, France

^3^Centre Georges François Leclerc, Dijon, France

***Correspondence :**

Ronan Quéré, Inserm/Université de Bourgogne, Dijon, France.

7, bd Jeanne d’Arc,

21000 Dijon, France

ronan.quere@inserm.fr

**Supplementary Figures S1 to S5**

**
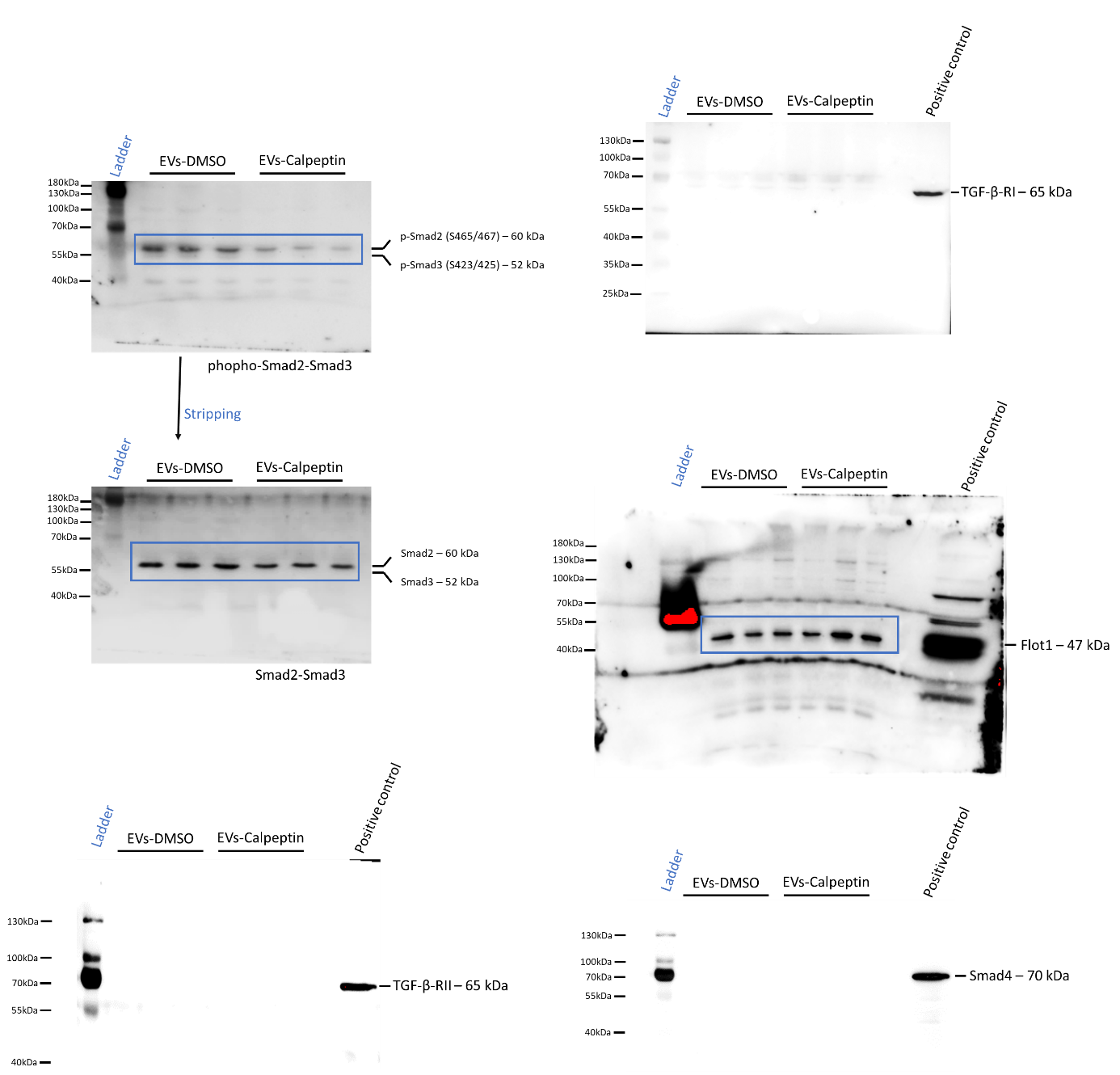
**

**Figure S1: Full-length uncropped Western blot corresponding to Fig. 1E.** Blues lines corresponding to cropped Western blots shown on Fig. 1E. Western blot also showing absence of the TGF-β receptors RI and RII, as well as Smad4 in EVs isolated *ex vivo* from BM. Positive control corresponds to MS-5 cell lysate.


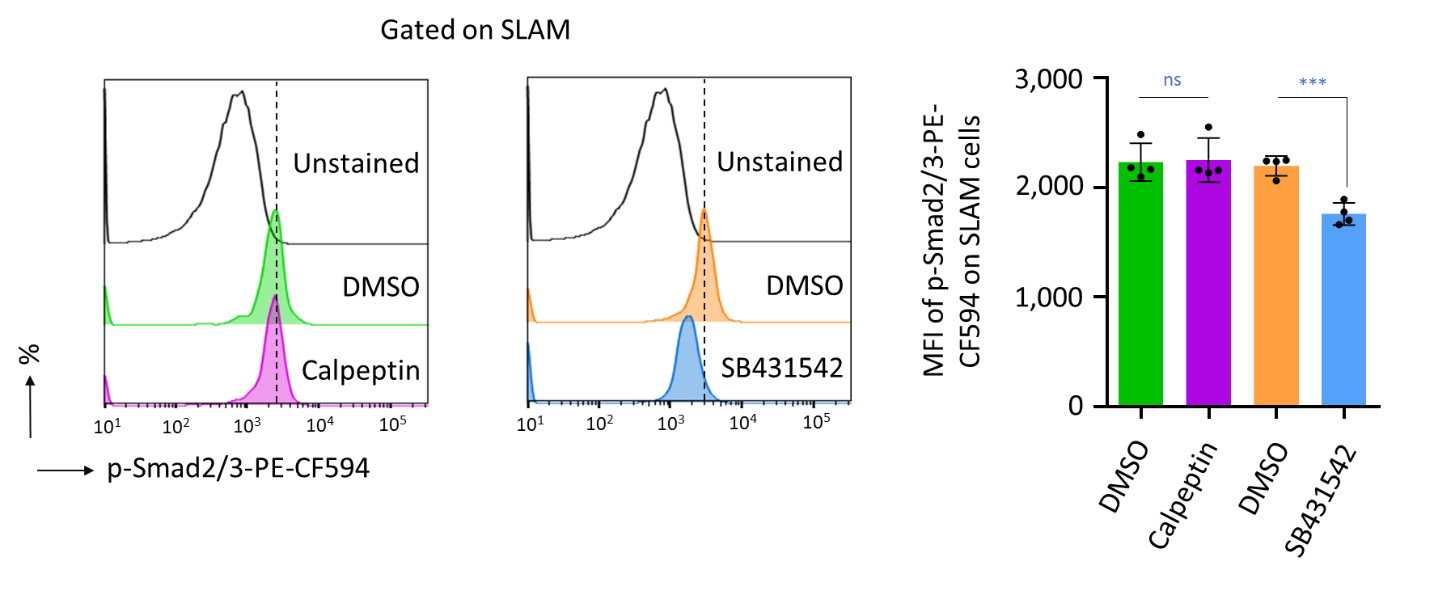


**Figure S2:** **Calpeptin is not an inhibitor of the TGF-β pathway.** Lin^-^ cells (2.5×10^5^ cells) isolated from mice were treated *ex vivo* with 0.5 µM of Calpeptin or 0.5 µM of the TGF-β-RI inhibitor SB431542. Following 18 hours, mean fluorescence intensity (MFI) measured by flow cytometry shows that the TGF-β inhibitor reduced the level of p-Smad2/3 on SLAM cells while Calpeptin did not. Data are shown as means ± SD; *n* = 4 biological replicates. *P* value measured by two-tailed unpaired Student’s *t* test; ****P* < 0.001; ns, non-significant.


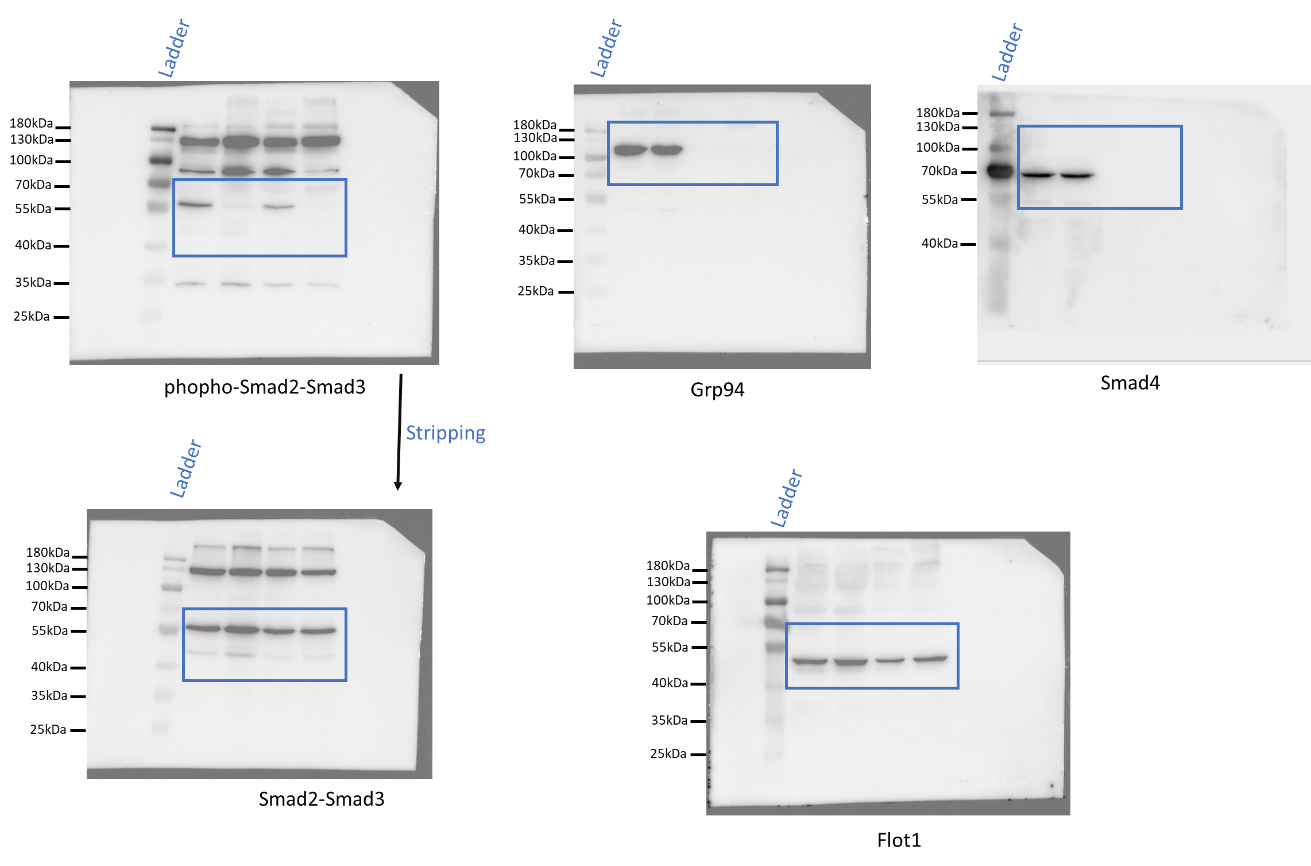


**Figure S3:** **Full-length uncropped Western blot corresponding to Fig. 3D.** Blues lines corresponding to cropped Western blots shown on Fig. 3D.

**
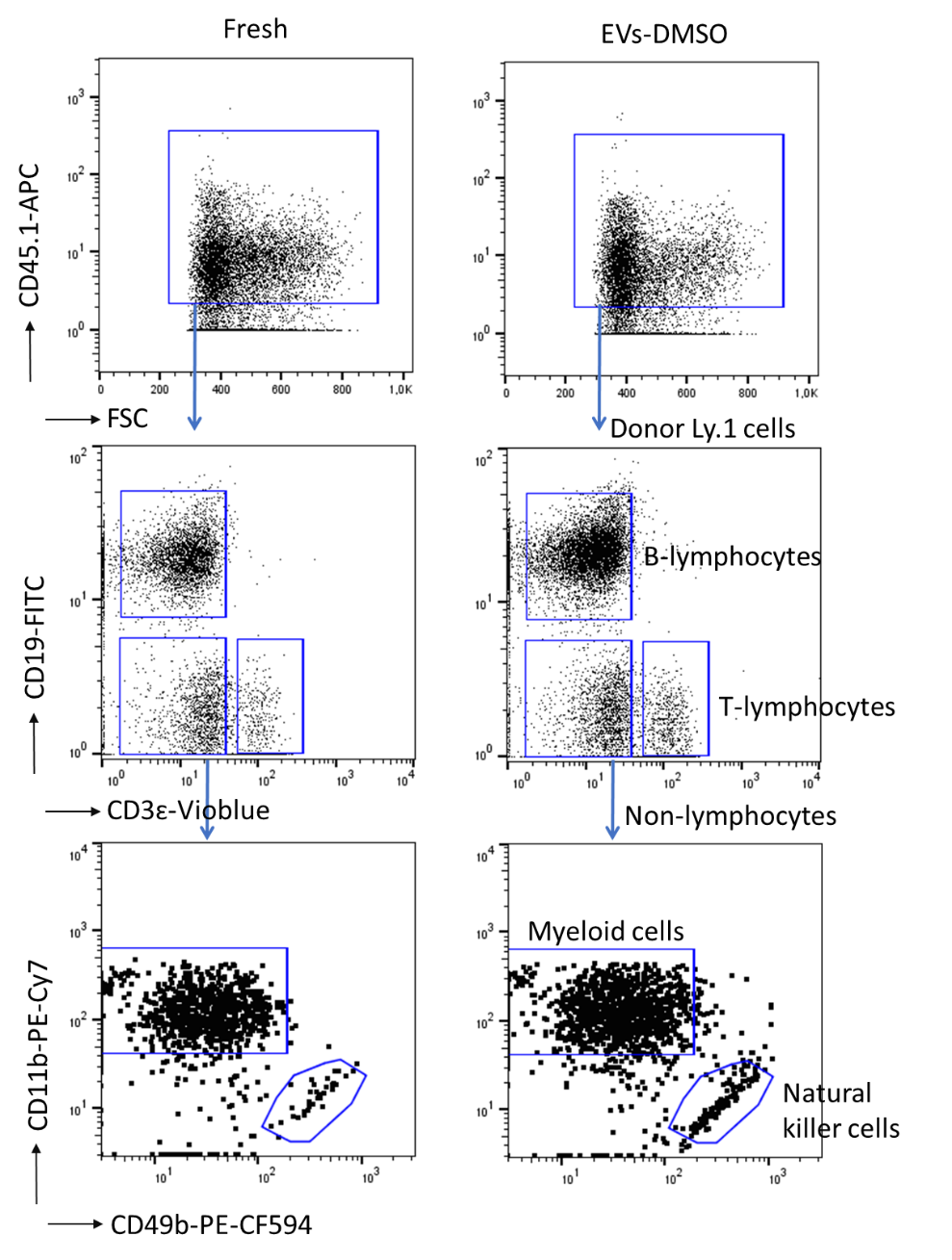
**

**Figure S4:** **Flow cytometry gating strategy corresponding to Fig. 6C.** Flow cytometry data recorded on viable (FVS780 negative) WBC in PB, 16 weeks after the transplantation of fresh Sca1^+^ cells or Sca1^+^ cells treated *ex vivo* with EVs-DMSO for 48 hours. Data were gated on the donor Ly.1 positive cells.

**
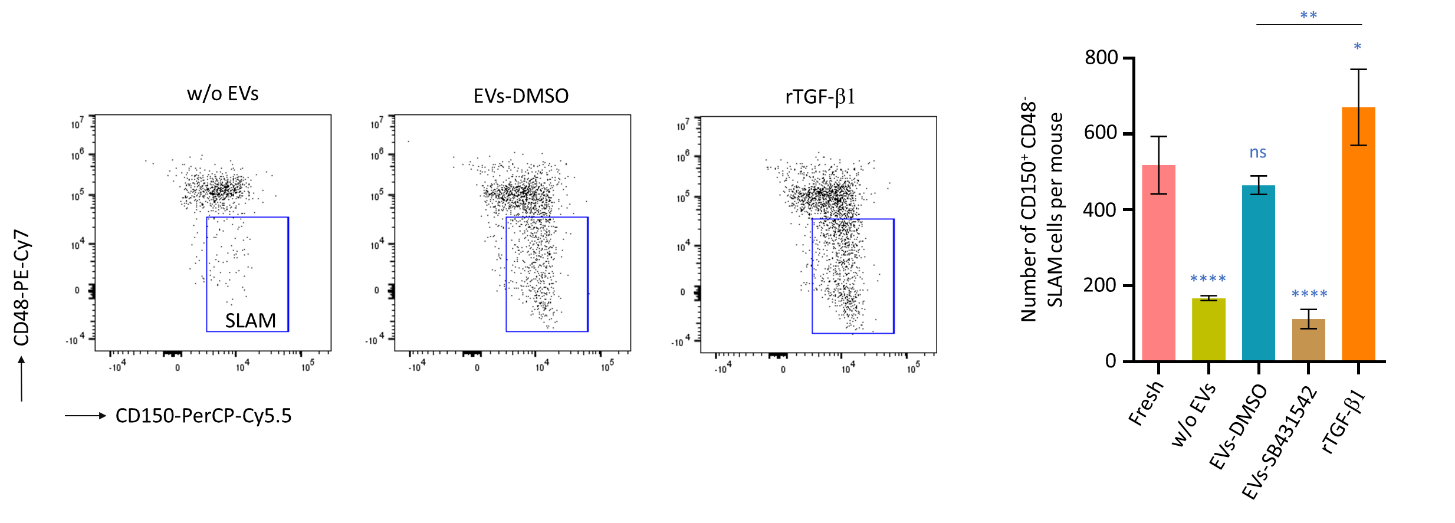
**

**Figure S5:** **Recombinant TGF-β1 ligand is more efficient than the use of EVs for an *ex vivo* maintenance of HSC.** Sca1^+^ HSC freshly (fresh) isolated *ex vivo* were co-cultured with EVs-DMSO, EVs-SB431542, without EVs (w/o EVs), or treated with the recombinant TGF-β1 ligand (rTGF-β1), used at 10 ng/mL. We administered 10^9^ particles per 4×10^5^ Sca1^+^ cells for 48 hours. Flow cytometry data recorded on viable cells (FVS440UV negative) and c-Kit^+^ Sca1^+^ cells. After 48 hours, the number of SLAM cells was assessed by flow cytometry. Data are shown as means ± SD; *n* = 4 biological replicates. *P* value calculated against the fresh condition and measured using one-way ANOVA with Tukey’s multiple comparison test; *P < 0.05; **P < 0.01; ****P < 0.0001; ns, non-significant.
